# Communicative patterns in online health communities: A comparative study of Italian and Polish

**DOI:** 10.1371/journal.pone.0333011

**Published:** 2025-09-23

**Authors:** Ramona Bongelli, Anna Tereszkiewicz, Marina Paolanti, Ilaria Riccioni

**Affiliations:** 1 Department of Political Sciences, Communication and International Relations, University of Macerata, Italy; 2 Institute of English Studies, Jagiellonian University, Krakow, Poland; 3 Department of Education, Cultural Heritage and Tourism, University of Macerata, Italy; School of Nursing Sao Joao de Deus, Evora University, PORTUGAL

## Abstract

This paper explores how online health platforms influence communication between patients and healthcare professionals, focusing on anxiety-related interactions in Italy and Poland. The study examines pairs of questions and answers, selected from Q&A services, within *MioDottore* and *ZnanyLekarz*, two medical platforms operating in these countries, with the main aim of identifying cross-cultural similarities and differences in communication strategies. Anxiety was chosen as a case study because it is one of the most prevalent mental health conditions, as revealed by research and national statistics, and because it is a prominent topic of discussion within online medical communities. Italy and Poland were chosen for comparison because of their different linguistic and socio-cultural contexts. Data were collected through an automated process based on a web-scraping algorithm and analysed using a mixed methods approach combining quantitative and qualitative techniques. The results show some clear differences in communicative patterns: Italian patients tend to use more indirect and polite forms, while Polish patients use a more direct style. Among practitioners, gender-related differences were found in the Polish responses: Polish women tend to adopt a more emphatic style. By contrast, Italian responses seem to be more influenced by professional specialisation: while psychiatrists tend to adopt a more pragmatic style (focusing more on the informational and request-oriented aspects of users’ posts), psychologists typically adopt a more empathic approach (paying more attention to the emotional dimension, often linked to the suffering experiences disclosed by users). These findings highlight how digital health communication reflects broader cultural and professional norms, although further research is needed to confirm these patterns across different conditions and clinical settings.

## 1. Introduction

Understanding how the Internet, digital technologies, and online services have changed relational and communicative models in healthcare is a complex challenge, particularly in cross-cultural contexts. The last few decades have introduced significant changes in this area, as in many other areas of life. With regard to health, the rise of the internet and social media, combined with technological advances, has led to

- the emergence of a wide array of devices, digital applications and patient-centric systems, which are designed not only to monitor patients’ health [1], but also to promote their engagement and empowerment (e.g., [2–3]), encouraging them to take a more active role in managing their own health [4];
- the possibilities - at least for some many medical specialties - to use telemedicine, which - once a few potential barriers have been overcome, such as those related to inadequate patient training [5] - makes it possible to break geographical barriers and improve access to healthcare (e.g., [6]), as well as to
- the increasing use of a large variety of healthcare-related services [7], which has allowed users not only to search for medical information - through online medical databases (e.g. [8–9]) and AI-driven medical chatbots (e.g., [10–11]) or more general ones (e.g., [12]) - but also to engage in peer-to-peer (i.e., peer-led) online communities. In this space, individuals - often patients or caregivers - share their personal experiences with illness, offer and receive emotional support, and exchange practical advice (e.g., [13–15]). Additionally, expert-based online communities allow users to ask questions directly to medical professionals and receive information, guidance, or second opinions based on clinical expertise (e.g., [16–17]).

In other words, from medical devices and telemedicine to AI-driven chatbots and online communities, these technologies are changing the way individuals seek and share information and how they receive care. This change is particularly significant when examined through an intercultural lens, as differences in language and, potentially, in digital literacy, or trust in medical institutions and attitudes towards health and technology can influence the way people use them.

This article adopts a comparative, cross-cultural perspective, focusing on the Italian and Polish contexts, and examines Q&A services commonly known as “Ask the Doctor” platforms [17], typically hosted within multi-service healthcare portals. These services have gained popularity as effective tools for user engagement and web traffic generation [18]. Two notable examples are the international portals *MioDottore* (Italy) and *ZnanyLekarz* (Poland), both part of one of the world’s largest healthcare platforms (https://www.docplanner.com, last accessed 9 May 2025), which operates in several European countries and beyond. The platform is aimed at individuals dealing with psychological, psychiatric, or physical conditions, as well as the general public, enabling users to submit health-related questions to professionals and receive timely information, opinions, advice, or support. In this article, the terms *patient* and *user* are used interchangeably to refer to individuals with a clinical condition who interact with healthcare professionals through online medical platforms. Similarly, the terms (*healthcare*) *expert*, *professional*, and *practitioner* are used as synonyms to refer to those providing health-related answers on these platforms.

While these online exchanges share certain epistemic similarities with traditional, in-person medical interactions, namely, the dynamic between a non-expert questioner and an expert responder, they also reflect several formal and relational differences. *Formally*, they are asynchronous, written, and mediated, and typically limited to a communicative activity, i.e., the exchange of questions and answers on a specific issue. In contrast, in-person interactions are synchronous, spoken, unmediated, and often involve a wider range of conversational activities over different topics. *Relationally*, these interactions take place between strangers: the practitioner does not know the patient’s medical history, and the patient does not know which professional from the platform will respond to their query. In contrast, face-to-face medical interactions take place normally between interlocutors with prior knowledge, except, of course, in the case of a first visit.

Extensive research has been conducted on peer-to-peer online healthcare communities (OHCs), including online health support groups (e.g., [19–23]), and more specific anxiety disorder online communities (ADOCs) (e.g., [24–25]). There have also been comparative studies. For example, Ardissone et al. (2024) [26] examined Italian and Polish OHCs in the context of diabetes management, highlighting the informative, supportive, and knowledge-compensatory functions of OHCs. In particular, they found that the compensation of essential knowledge, which is crucial for effective self-management, was partly achieved through peer-to-peer exchanges within the communities.

Moreover, although a substantial body of work also exists on OHCs involving user-expert interactions, i.e., patient-professional interactions (e.g., [17,27–30]), few studies have explored how mental disorders, such as *anxiety disorders*, are addressed in such online health communities (e.g. [31]). Furthermore, to the best of our knowledge, no study has yet adopted a comparative approach focusing on similarities and differences in communicative patterns, particularly in terms of pragmatic and empathic alignment.

To address this gap, we developed an automated process based on a web-scraping algorithm to extract data from one of the largest healthcare platforms operating in both Italy and Poland. This algorithm is specifically designed to collect question-answer pairs from patient-doctor interactions, with a particular focus on the initial responses provided by healthcare professionals.

Using this technology, we have built Italian and Polish corpora of data on anxiety disorders, which allow a comparative analysis of interactions from both pragmatic and empathic perspectives. The use of this advanced scraping methodology not only ensures efficient and accurate data collection, but also enables the acquisition of a large and diverse dataset, an essential prerequisite for studying the complex communicative dynamics within OHCs.

In other words, this study adopts a comparative approach to explore the communicative patterns of interaction in Italian and Polish Q&A forums, with the overarching goal of identifying differences and similarities. More specifically, it aims to investigate how users express anxiety-related concerns pragmatically and emotionally, and how practitioners reply to these concerns, in two culturally and linguistically distinct contexts. This research addresses the following questions:

RQ1: *What are the main pragmatic, politeness-related, and emotional features of questions posed by Italian and Polish users suffering from anxiety disorders in medical Q&A forums?*

RQ2: *What are the main pragmatic, politeness-related, and emotional features of Italian and Polish practitioners’ replies? Are they pragmatically and empathically aligned with the users’ messages?*

RQ3: *To what extent do response length, use of politeness features, and pragmatic and empathic alignment vary according to the gender and professional specialisation of the practitioners, and how do these patterns differ between the Italian and Polish data sets?*

## 2. Materials and methods

### 2.1 Data collection and extraction

Data collection was conducted in October 2023 for both the Italian and Polish languages, using “MioDottore” (Italy) and “ZnanyLekarz” (Poland), both part of one of the world’s largest healthcare platforms (https://www.docplanner.com, last accessed 9 May 2025).

Anxiety disorder was selected as the focus of this study due to its relevance within the broader domain of psychological conditions.

In the Italian context, data from the *Istituto Nazionale di Statistica* (ISTAT9 [National Institute of Statistics] indicate that, in 2023, 4.7% of the population reported suffering from nervous disorders. Additionally, according with the 2023 BES report (*Benessere Equo e Sostenibile in Italia*/ Fair and sustainable wellbeing in Italy, available at https://www.istat.it/wp-content/uploads/2024/04/Bes-2023-Ebook.pdf, last accessed 4 August 2025) the mental health index – measured through the SF-36 (36-Item Short Form Survey), which assesses four key dimensions of mental health: anxiety, depression, emotional or behavioural control, and psychological well-being – remained relatively stable in 2023 (68.7) compared to 2022 (69.0) and 2019 (68.4). This index ranges from 0 to 100, with higher scores indicating better psychological health. Despite this apparent stability, however, a worrying decline in psychological well-being has been observed since 2020, particularly among younger individuals, with adolescent girls being the most affected group.

As for Poland, according to the “State of Health in the EU Poland Country Health Profile 2023” (available at https://www.oecd.org/content/dam/oecd/en/publications/reports/2023/12/poland-country-health-profile-2023_80434439/f597c810-en.pdf, last accessed 4 August 2025), there are no systematic epidemiological studies of mental health disorders in the general population. Moreover, although the estimates from the Institute for Health Metrics and Evaluation (IHME) indicate that in 2019, 14% of people in Poland reported having a mental health problem (a percentage lower than the EU average of 17%), this rate is probably underestimated due to lower awareness of mental illness, stronger stigma, and more limited access to mental health services, which can reduce the chances of getting a diagnosis. Nonetheless, according to the reports “Health and healthcare in 2023” by Statistic Poland (available at https://stat.gov.pl/en/topics/health/health/health-and-health-care-in-2023,1,16.html, last accessed 4 August 2025), 4.3% more people than in 2021 were provided with outpatient psychiatric care in 2022.

Furthermore, anxiety emerged as one of the most frequently searched topics on the medical online platforms such as “MioDottore” and “ZnanyLekarz”, and was among the few conditions for which at least 100 Q&A pairs were available in both the Italian and Polish datasets.

While patients’ posts are completely anonymous on the platform (unless users choose to reveal their identity), doctors’ posts are not. Their name and area of specialisation are visible. However, in fragments presented in section 4 (*Some linguistic examples*), we anonymised both users’ and health professionals’ posts (consistently with the ethical statement presented at the end of the paper).

Data extraction was performed using an advanced web scraping system in a Linux environment designed to manage complex web structures. Python and the Selenium library (https://www.selenium.dev/documentation/) automated browser navigation on the DoctorPlanner website to retrieve dynamic JavaScript-rendered content. Custom scripts collected 100 anxiety-related question-answer pairs for Italian and Polish datasets. Bash scripts supported the automation and scheduling of scraping tasks to ensure efficiency. BeautifulSoup (https://pypi.org/project/beautifulsoup4/) was then used to parse and structure the raw HTML/XML data into a clean, analysable format. The data was stored in CSV files for durability and compatibility with analysis tools. To ensure high data quality, the scraping process was fine-tuned to capture only the first, reliable responses from healthcare professionals to users’ questions.

### 2.2 Procedures

To answer the RQs, a preliminary labelling of the question-answer pairs was carried out.

#### 2.2.1 Tagging steps.

The tagging process for both questions and answers in the two corpora was carried out manually by the authors, with close attention to detail. The procedure involved several key stages to ensure accuracy and consistency. First, the authors carried out a joint preliminary analysis of both corpora to identify their main features. Based on this, they jointly defined a set of categories and developed a detailed coding grid to guide the annotation process.

The corpora were then tagged individually. Interobserver agreement was assessed using Cohen’s kappa (κ), calculated separately for each annotation category. For the Italian corpus, agreement was measured between two of authors (BR and IR); for the Polish corpus, between one of the authors (TA) and one of her graduate students, who assisted with the tagging process. Cohen’s κ ranged, among different categories, from 0.83 to 1 for the Italian corpus, and from 0.783 to 1 for the Polish one. After calculating agreement scores, the coders met regularly to discuss ambiguous or discordant cases and reach consensus through collaborative decision-making. Once disagreements were resolved and coding consistency was ensured, the dataset was finalized for subsequent quantitative and statistical analysis.

#### 2.2.2 Tagging.

User posts were first categorized based on the presence or absence of questions and/or requests. Posts containing one or more questions or requests were further tagged according to their form, i.e., they were classified as direct, indirect, implicit, or mixed, as well as according to their main pragmatic functions, i.e., they were classified as: request for information/opinion/confirmation; request for advice/help/reassurance; venting/disclosure. Additionally, all users’ posts were also classified based on the presence or absence of conversational politeness features: greetings, forms of address (appellations), and acknowledgements, as well as on the basis of the presence or absence of emotional expressions (i.e., expressions emotionally characterised).

Health professionals’ replies were categorized based on the professional background of the responder (primarily psychiatrists and psychotherapists), and similarly tagged for the presence of greetings, appellations, acknowledgements, and expressions of encouragement/best wishes/declaration of availability, all politeness strategies that, according to Zhao and Mao (2019) [32], are specifically used in OHCs to create a comfortable communicative atmosphere, demonstrate respect for patients, promote positive feelings toward them, and build a relationship of trust. The replies were also classified based on the presence or absence of emotional expressions. Finally, pragmatic and empathetic alignment was assessed, understood as a form of accommodation to users’ information needs and requests for empathic understanding. “Medical empathy has been defined as the predominantly cognitive attribute that involves the ability to understand patient’s experiences, concerns, and perspectives, and communicate this understanding with the intention of helping” ( [33], p. 154).

The categorization criteria used for tagging both corpora – the Italian Anxiety Corpus (IAC) and the Polish Anxiety Corpus (PAC) – are detailed in S1Table.

#### 2.2.3 The analysis.

Following the tagging process, quantitative and statistical analyses were conducted using version 2.3.28 of *Jamovi*, an open-source statistical software (retrieved from https://www.jamovi.org), built on top of the R statistical language (retrieved from https://cran.r-project.org), with R packages sourced from the MRAN snapshot dated 2022-01-01. The primary objectives of these analyses were to identify the main characteristics of both corpora and to compare their communicative patterns.

The quantitative results discussed below are followed by a content pragma-linguistic analysis of a selection of question–response pairs (see section 4), aiming to illustrate some specific patterns identified in both corpora.

## 3. Results

Before presenting the main quantitative findings from the frequency and inferential analyses, we provide an overview of the key characteristics of both corpora in the Table 1 below.

**Table 1:** Corpora overview and healthcare professionals’ characteristics in IAC and PAC.

	IAC		PAC	
	Freq	%	Freq	%
**Corpora dimensions**				
*Words count*				
Total words of users’ posts	11814	68.32%	9111	62.47%
Total words of experts’ posts	5478	31.68%	5473	37.53%
** Tot.**	**17292**	100%	**14584**	100%
*Means words for posts*				
Users	118		91.42	
Healthcare professionals	55		54.8	
**Socio-demo characteristics of healthcare professionals**				
*Gender*				
Male	69	69%	67	67%
Female	31	31%	33	33%
** Tot.**	100	100%	100	100%
*Job specialisations/roles*				
Psychiatrist	36	36%	42	42%
Psychiatrist and psychotherapist	21	21%	4	4%
Psychologist and psychotherapist	25	25%	51	51%
Other medical practitioners	18	18%	3	3%
** Tot.**	100	100%	100	100%

As for corpora dimensions, IAC is slightly bigger, with a higher total number of words of users’ posts, and a higher share of these posts in comparison to PAC. The mean number of words per post in the case of users’ messages is higher in IAC, while the mean number of words per post in the case of experts’ messages is almost identical in both corpora.

As far as socio-demographic characteristics of the healthcare professionals are concerned, the gender distribution is similar in both corpora, with a significantly higher proportion of males (69% in IAC and 67% in PEC).

Differences between IAC and PAC can be seen in practitioners’ professional specialisations. While the proportion of psychiatrists is relatively similar in both corpora (36% in IAC, 42% in PAC), the number of psychiatrists with the specialization (i.e., with a training) in psychotherapy is significantly higher in IAC than in PAC. In PAC, by contrast, the number of experts specialising in psychology and psychotherapy is noticeably higher than in IAC. The corpora also differ in the proportion of experts in other medical specialisations (e.g. surgery, gynaecology), which is significantly higher in IAC than in PAC. Although each corpus contains 100 pairs of questions and answers, it should be noted that these responses were not provided by 100 distinct practitioners, as some experts may have answered more than one question. The total number of experts is 49 in IAC and 55 in PAC.

Results of the quantitative analysis are presented in the following sections.

### 3.1 Quantitative results for Italian and Polish Anxiety Corpora

The main quantitative results concerning the features of patients’ posts, practitioners’ replies, and empathic and pragmatic alignment in both IAC and PAC are shown in Table 2. In addition to significance values, effect size measures were included wherever possible to assess the strength of these associations. In this case, Cohen’s w was used to measure effect size (w = .10, small effect; w = .30, medium effect; w = .50, large effect [34]).

**Table 2:** Distribution and statistical results for IAC and PAC online anxiety consultation corpora.

	IAC			PAC		
	Freq	%	χ² and Cohen’s w for the effect size	Freq	%	χ² and Cohen’s w for the effect size
**PATIENTS**						
***Types of questions***			χ² (4) = 228, p = < .001** w = 1.25, indicating a very large effect			χ² (4) = 369, p = < .001** w = 1.57, indicating a very large effect
direct question/request	98	66.7 %		123	83.1 %	
indirect question/request	37	25.2 %		3	2.0 %	
mixed form	5	3.4 %		5	3.4 %	
no_question	4	2.7 %		7	4.7 %	
implicit question/request	3	2.0 %		10	6.8 %	
**Tot.**	**147**	100%		**148**	100%	
***Questions pragmatic functions***			χ² (2) = 86.5, p = < .001** w = 0.76, indicating a very large effect			χ² (2) = 78.8, p = < .001** w = 0.72, indicating a very large effect
requests for information/opinion/confirmation	96	65.3%		95	64.2%	
requests for help/advice/reassurance	47	32.0%		46	31.1%	
venting/disclosure	4	2.7%		7	4.7%	
**Tot.**	147	100%		148	100%	
***Greetings/Salutations***			χ² (1) = 41.0, p = < .001** w = 0.64, indicating a very large effect			χ² (1) = 4.84, p = 0.028* w = 0.22, indicating a small to medium effect
Present	82	82%		39	39.0%	
Absent	18	18%		61	61.0%	
**Tot.**	100	100%		100	100%	
***Appellations/Titles***			χ² (1) = 67.2, p = < .001** w = 0.81, indicating a very large effect			–
Present	9	91%		0	0%	
Absent	91	9%		100	100%	
**Tot.**	100	100%		100	100%	
***Acknowledgments***			χ² (1) = 1.44, p = 0.230			χ² (1) = 57.8, p = < .001** w = 0.76, indicating a very large effect
Present	56	56%		12	12%	
Absent	44	44%		88	88%	
**Tot.**	100	100%		100	100%	
***Emotional components***			χ² (1) = 11.6, p = < .001** w = 0.34, indicating a medium to large effect			χ² (1) = 14.4, p = < .001** w = 0.37, indicating a medium to large effect
Present	67	67%		69	69%	
Absent	33	33%		31	31%	
**Tot.**	100	100%		100	100%	
**HEALTHCARE PRACTITIONERS**						
***Greetings/Salutations***			χ² (1) = 16.0, p = < .001** w = 0.4, indicating a medium to large effect			χ² (1) = 9.00, p = 0.003* w = 0.3, indicating a medium effect
Present	70	70%		65	65%	
Absent	30	30%		35	35%	
**Tot.**	100	100%		100	100%	
***Appellations/Titles***			χ² (1) = 74.0, p = < .001** w = 0.86, indicating a very large effect			χ² (1) = 51.8, p = < .001** w = 0.71, indicating a very large effect
Present	7	7%		14	14%	
Absent	93	93%		86	86%	
**Tot.**	100	100%		100	100%	
***Acknowledgments***			–			–
Present	0	0%		0	0%	
Absent	100	100%		100	100%	
**Tot.**	100	100%		100	100%	
***Expressions of encouragement and/or best wishes, declarations of availability***			χ² (1) = 36.0, p = < .001** w = 0.6, indicating a very large effect			χ² (1) = 38.44, p = < .001** w = 0.62, indicating a very large effect
Present	20	20%		19	19%	
Absent	80	80%		81	81%	
**Tot.**	100	100%		100	100%	
***Emotional components***			χ² (1) = 21.2, p = < .001** w = 0.46, indicating a medium to large effect			χ² (1) = 36.0, p = < .001** w = 0.6, indicating a very large effect
Present	27	27%		20	20%	
Absent	73	73%		80	80%	
**Tot.**	100	100%		100	100%	
**EMPATHIC ALIGNMENT**						
Alignment	51	51%	χ² (1) = 0.0400, p = 0.841	51	51%	χ² (1) = 0.0400, p = 0.841
Misalignment	49	49%		49	49%	
**Tot.**	100	100%		100	100%	
**PRAGMATIC ALIGNMENT**						
Alignment	89	60.5%	χ² (2) = 62.2, p = < .001** w = 0.65, indicating a very large effect	80	54.1%	χ² (2) = 39.0, p = < .001** w = 0.51, indicating a large effect
Misalignment	47	32.0%		50	33.8%	
Partial alignment	11	7.5%		18	12.2%	
**Tot.**	147	100%		148	100%	

#### 3.1.1 Italian Anxiety Corpus: features of patients’ posts, characteristics of practitioners’ responses, and patterns of pragmatic and empathic alignment.

As for IAC, of 100 users’ posts, only 4 did not include any questions. Of the remaining 96 posts, 143 questions/requests were identified, the most numerous of which are direct, followed by indirect, mixed and finally by implicit forms. These differences are statistically significant (χ² (4) = 228, p = < .001**, Cohen’s w = 1.25, indicating a very large effect) and are due to the contribution of all classes, except for indirect questions. While the number of direct questions/requests is higher than expected (R = 12.65), the observed frequencies for the mixed form, non-questions and implicit questions/requests are lower than expected (respectively, R = −4.68; −4.86; −4.50). As for the pragmatic functions, most question/requests are used to ask for information, opinions, or confirmation of one’s assumptions. A share of 32% consists of requests for advice, help, or reassurance, and finally, there are only 4 occurrences classified as venting or disclosure. The difference in the use of these types of questions is statistically significant (χ² (2) = 86.5, p = < .001**, Cohen’s w = 0.76, indicating a very large effect) and can be attributed to a higher-than-expected number of requests for information, opinions, or confirmation (R = 6.71), and to a lower-than-expected number of instances of venting or disclosure (R = −6.42). The majority of users’ posts include greetings, acknowledgements and emotional components. In contrast, appellations are present in only a few posts.

The situation is quite different when it comes to practitioners’ posts. In most cases, they greet the users who asked the questions, but expressions of acknowledgement (e.g., thanking them for reaching out) are entirely absent. Only 7 out of 100 replies include appellations. In a rather low percentage, practitioners use expressions of encouragement, good wishes and declarations of helpfulness. Specifically, such expressions appear in 20% of their posts. Regarding emotional components, the trend appears to be the opposite of what we observed for patients: 27 posts include emotional elements, while 73 do not.

As for alignment, while no statistically significant differences were found for empathic replies (with 51 aligned and 49 misaligned responses), significant differences emerged for pragmatic replies (χ² (2) = 62.2, p = < .001**, Cohen’s w = 0.65, indicating a very large effect). In this case, fully aligned replies were more frequent than expected (R = 5.71), whereas misaligned and partially aligned replies were less frequent than expected (R = −0.28 and R = −5.42, respectively).

#### 3.1.2 Polish Anxiety Corpus: features of patients’ posts, characteristics of practitioners’ responses, and patterns of pragmatic and empathic alignment.

As for PAC, of 100 users’ posts, 7 did not include any questions. Of the remaining 93 posts, 141 questions/requests were identified, with direct forms being the most numerous, followed by implicit, mixed and finally by indirect forms. These differences are statistically significant and are due to the contribution of all classes (χ² (4) = 369, p = < .001**, Cohen’s w = 1.57, indicating a very large effect). While the number of direct questions/requests is higher than expected (R = 17.16), the observed frequencies for the implicit, non-questions, mixed forms and indirect questions/requests are lower than expected (respectively, R = −3.60; −4.15; −4.52; −4.88). As for the pragmatic functions most question/requests are used to ask for information, opinion or confirmation. A share of 31% consists of requests for advice, help or reassurance, and finally there are only 7 occurrences classified as venting or disclosure. The difference in the use of these types of questions is statistically significant (χ² (2) = 78.8, p = < .001**, Cohen’s w = 0.72, indicating a very large effect) and can be attributed to a higher-than-expected number of requests for information, opinions, or confirmation (R = 6.50), and to a lower-than-expected number of instances of venting or disclosure (R = –6.02). The majority of users’ posts include emotional components. There are few instances of greetings and acknowledgements, while appellations are absent. Expressions of encouragement, wishes, declarations of availability are present in 19% of the posts.

The situation is quite different for healthcare professionals’ posts. In most cases, they greet the users who asked the questions, but expressions of acknowledgment (e.g., thanking them for reaching out) are completely absent. Only 14 out of 100 replies include appellations. A rather low percentage of them use expressions of encouragement, good wishes and declarations of helpfulness. Regarding emotional components, the trend appears to be the opposite of what we observed with patients: 20 posts include emotional elements, while 80 do not.

As for alignment, while no statistically significant differences were found for empathic replies (with 51 aligned and 49 misaligned responses), significant differences emerged for pragmatic replies (χ² (2) = 39.0, p = < .001**, Cohen’s w = 0.51, indicating a large effect). In this case, fully aligned replies were more frequent than expected (R = 6.5), whereas partially aligned replies were less frequent than expected (R = −3.36).

#### 3.1.3 Comparative results: patients’ posts, practitioners’ replies, and alignment patterns in IAC vs. PAC.

The main differences between IAC and PAC are summarized below.

A more frequent use of direct questions and implicit requests and less frequent use of indirect questions (often more moderated) can be observed in PAC than in IAC. As for the pragmatic functions, the two corpora are almost identical, with a similar share of requests for information, opinion or confirmation and requests for help or advice. There is a slightly higher frequency of posts classified as venting or disclosure in PAC. While in IAC most patients use greetings, less than half do so in PAC. Similarly, expressions of thanks are present in more than half of the posts in IAC, but only in 12% of the posts in PAC. As for appellations, their presence is low in IAC (9%), and in PAC they are completely absent. Despite these formal differences, there are similarities in the presence of emotional components which can be interpreted as explicit or implicit requests for emotional support. These are present in over 60% of patients’ posts in both IAC and PAC.

In contrast to the patients’ posts, the practitioners’ posts show a higher degree of similarity between the two corpora. In both IAC and PAC, doctors greet patients in the majority of cases (70% in IAC, 65% in PAC). However, there are no expressions of thanks towards the patients in either corpus. Forms of appellation are rarely used in IAC and are entirely absent in PAC. In both corpora, the percentage of practitioners using expressions of encouragement, good wishes and declarations of helpfulness is around 20%. Emotionally connoted language appears in less than 30% of practitioners’ posts (27% in PAC, 20% in IAC).

Finally, regarding *empathic alignment*, the situation is almost identical in both IAC and PAC, with the same proportion of aligned and misaligned cases. As for *pragmatic alignment*, there is a slight difference: the IAC corpus shows a marginally higher percentage of alignment compared to the PAC corpus.

#### 3.1.4 In-depth analysis of empathic alignment.

As for empathic alignment, four possible interaction scenarios were identified based on the presence or absence of emotional requests and the alignment/misalignment of the response:

1. Emotional request with empathically aligned response
2. Emotional request with empathically misaligned response
3. No emotional request with non-empathic but appropriately aligned response
4. No emotional request with empathically misaligned (unsolicited) response

In Tables 3 and 4, the main quantitative results concerning empathic alignment in IAC and PAC are presented.

**Table 3:** Emotional components and empathic alignment in IAC and PAC.

Contingency Tables								
	Empathic alignment IAC				Empathic alignment PAC			
Emotional components	misalignment	alignment	Tot.	χ², Phi-coefficient and Cramer’s v	misalignment	alignment	Tot.	χ², Phi-coefficient and Cramer’s v
Present	45	22	67	χ² (1) = 26.8, p = < .001** φ = 0.518 v = 0.518	49	20	69	χ² (1) = 43.2, p = < .001** φ = 0.657 v = 0.657
Absent	4	29	33		0	31	31	
**Tot.**	**49**	**51**	100		**49**	**51**	100	

**Table 4:** Types of alignment to patient emotional components in IAC and PAC.

		IAC	PAC
Patient	Doctor	Freq	Freq
Emotional request	Empathically aligned response	22	20
Emotional request	Empathically misalignment response	45	49
No emotional request	Non-empathic but aligned response	29	31
No emotional request	Empathic misaligned (unsolicited) response	4	0
Tot.		**100**	100

#### IAC.

As for empathic alignment, as shown in Table 3, out of the 67 users’ posts with emotional components, 45 are not empathically aligned, while only 22 are. Of the 33 users’ posts without emotional components, 29 replies are aligned (i.e., they avoid empathically connoted replies) and 4 are not (i.e., they involve empathically connoted responses to requests lacking emotional components or requests to be supported emotionally).

In other words, while most emotional users’ posts are not empathically aligned (45), most non-emotional posts are aligned (29).

There is a strong counterintuitive association (χ² (1) = 26.8, p = < .001*) between the presence of emotional components and the type of empathic alignment: posts with emotional components tend to be less empathically aligned, while ‘colder’ posts (without emotions) are more often aligned.

The strength of this association, as indicated by both the phi coefficient and Cramér’s V, is substantial (φ and V = 0.518), suggesting a large effect size.

#### PAC.

As for empathic alignment, as shown in Table 4, out of the 69 users’ posts with emotional components, 49 are not empathically aligned, while only 20 are. Of the 31 users’ posts without emotional components, all replies are aligned (i.e., they avoid empathically connoted replies). In other words, while most of the emotional patients’ posts are not empathically aligned, all of the non-emotional posts are aligned.

There is therefore also a strong counterintuitive association (χ² (1) = 43.2, p = < .001*) between the presence of emotional components and the type of empathic alignment: posts with emotional components tend to be less empathically aligned, while ‘colder’ posts (without emotions) are always aligned. The effect size, measured by both the phi coefficient and Cramér’s v, is equal to 0.657, indicating a large effect.

#### Comparison.

In the PAC corpus, misalignment typically occurs with patients’ posts that contain emotional components that function as explicit or implicit requests for emotional support. In these cases, the expert bypasses the emotional content of the patient’s post. In contrast, in the IAC corpus, although only in four cases, misalignment does not exclusively follow posts with emotional requests that are ignored by the practitioner. Instead, it can occur as an emotionally charged but misaligned response to a request that does not contain any emotional element that could be interpreted as a request for support.

Empathic alignments following emotionally connoted request posts are similar in both corpora, as are non-empathic alignments following requests that lack emotional content.

#### 3.1.5 Politeness strategies, response length, and alignment: effects of gender and job specialisation on practitioners’ replies.

T-test, ANOVA and contingency table analyses were conducted to assess whether practitioners’ gender and job specialisation influenced the length of responses, the use of politeness-related features, as well as pragmatic and empathic alignments.

#### IAC.

As for the length of responses, the independent samples t-test (assuming unequal variances due to a significant Levene’s test) showed that the difference in the number of words used by male and female doctors was not statistically significant, *t*(98.0) = –1.08, *p* = .281. In contrast, the ANOVA revealed significant differences in the number of words used by practitioners with different specialisations, *F*(3, 42.0) = 14.4, *p* < .001. As shown in Table 5, the Tukey post-hoc test revealed that psychologists and psychotherapists used significantly more words than all other groups: the difference was significant when compared to other medical practitioners (*p* = .012), psychiatrists (*p* < .001), and psychiatrists with psychotherapeutic training (*p* < .001).

**Table 5:** Differences in response lengths among practitioners with different job specialisations.

		Other medical practitioners	Psychiatrist	Psychiatrist and psychotherapist	Psychologist and psychotherapist
Other medical practitioners	Mean difference	—	22.0	24.67	−33.9*
	p-value	—	0.138	0.132	0.012
Psychiatrist	Mean difference		—	2.69	−55.8***
	p-value		—	0.992	< .001
Psychiatrist and psychotherapist	Mean difference			—	−58.5***
	p-value			—	< .001
Psychologist and psychotherapist	Mean difference				—
	p-value				—

*Note.* * p < .05, ** p < .01, *** p < .001.

As for the politeness-related features, contingency table analyses revealed no significant gender-based differences in the use of greetings (χ²(1, N = 100) = 0.38, p = .540), appellations (χ²(1, N = 100) = 0.021, p = .885), as well as expressions of encouragement and/or best wishes and declarations of availability (χ²(1, N = 100) = 0.187, p = .665). Similarly, no statistically significant gender-based differences were found for pragmatic alignment (χ²(2, N = 147) = 3.84, p = .147), although female practitioners demonstrated a higher rate of alignment in their responses (70.5% of 44) compared to male experts (56.3% of 103). No statistical difference related to gender was observed for empathic alignment (χ²(1, N = 100) = 0.265, p = 0.607).

In contrast to gender, practitioners’ job specialisation appears to lead to a significantly different use of politeness formulae. Specifically, the contingency table analysis revealed statistically significant differences in the use of greetings (χ²(3, N = 100) = 15.0, p = .002, Cramer’s v = 0.387, indicating a medium to large effect), with psychologists and psychotherapists employing such politeness formulae almost consistently (24 out of 25 cases), whereas psychiatrists used them in only half of the cases (18 out of 36), and psychiatrists with psychotherapy training in more than 70% of the cases (15 out of 21) (see Table 6).

**Table 6:** Use of greetings by practitioners according to job specialisations.

	Practitioners’ job specialisations				
Practitioners Greetings	Other medical practitioners	Psychiatrist	Psychiatrist and psychotherapist	Psychologist and psychotherapist	Tot.
Present	13 (72.2%)	18 (50%)	15 (71.4%)	24 (96%)	70 (70%)
Absent	5 (27.8%)	18 (50%)	6 (28.6%)	1 (4%)	30 (30%)
**Tot.**	18 (100%)	36 (100%)	21 (100%)	25 (100%)	100 (100%)

Statistically significant differences were also observed in the use of appellations (χ²(3, N = 100) = 9.98, p = .019, Cramer’s v = 0.316, indicating a medium to large effect). The 7 cases identified were used only by other doctors (3 occurrences out of 18) and by psychologists and psychotherapists (4 occurrences out of 21). All the other practitioners did not use them (see Table 7).

**Table 7:** Use of appellations by practitioners according to job specialisations.

	Practitioners’ job specialisation				
Practitioners Appellations	Other medical practitioners	Psychiatrist	Psychiatrist and psychotherapist	Psychologist and psychotherapist	Tot.
Absent	15 (83.3%)	36 (100%)	21 (100%)	21 (84%)	93 (93%)
Present	3 (16.7%)	0 (0%)	0 (0%)	4 (16%)	7 (7%)
**Tot.**	18 (100%)	36 (100%)	21 (100%)	25 (100%)	100 (100%)

Contingency table analysis also revealed a significant association between job specialisation and the use of expressions of encouragement, best wishes, or declarations of availability (χ²(3, N = 100) =40.9, p= < .001; Cramer’s v = 0.639, indicating a very large effect). While such expressions were absent in posts by other medical professionals and were rarely used by psychiatrists and psychiatrists with psychotherapy training, they were frequently employed by psychologists and psychotherapists (16 cases out 25) (see Table 8).

**Table 8:** Use of encouragement best wishes, declarations of availability by practitioners according to job specialisations.

	Practitioners’ job specialisation				
Practitioners’ expressions of encouragement and/or best wishes, declarations or availability	Other medical practitioners	Psychiatrist	Psychiatrist and psychotherapist	Psychologist and psychotherapist	Tot.
Absent	18 (100%)	34 (94.4%)	19 (90.5%)	9 (36%)	80 (80%)
Present	0 (0%)	2 (5.6%)	2 (9.5%)	**16** **(64%)**	20 (20%)
Tot.	18 (100%)	36 (100%)	21 (100%)	25 (100%)	100 (100%)

Although some differences in pragmatic alignment emerge across professional categories, with medical professionals (psychiatrists with psychotherapeutic training, psychiatrists, and other medical practitioners) showing higher alignment (21 out of 32 replies; 35 out of 54; 15 out of 22, respectively) than psychotherapists (18 out of 39), the association was not statistically significant (χ²(6) = 9.80, p = .133), suggesting that profession type alone may not be a strong predictor of alignment in this sample As for the empathic alignment, although the contingency table analysis did not reveal a statistically significant association (χ²(3, N = 100) = 6.18, p = .103), nonetheless, psychologists and psychotherapists provided more often aligned replies (18 out of 25) than other practitioners.

#### PAC.

As for PAC, the independent samples t-test revealed that the difference in the number of words used by male and female doctors was not statistically significant *t*(97) = 0.708, *p* = .481. To assess whether professional specialisation also affected the number of words used by practitioners in the PAC dataset, a non-parametric test (Kruskal-Wallis) was used instead of ANOVA, due to the large differences in group sizes and the particularly small sample sizes in two categories (psychiatrists with psychotherapeutic training and other doctors). The test revealed some significant differences (χ²(3, N = 100) = 30.8, p = < .001). Specifically, as shown in Table 9, psychologists and psychotherapists used significantly more words than doctors representing other medical specialisations. It was also found that psychiatrists used significantly fewer words than psychologists and psychotherapists.

**Table 9:** Word count comparisons across practitioners’ job specialisations.

Pairwise comparisons – numbers of words			
		W	p
Other medical practitioners	Psychologist and psychotherapist	3.981	**0.025**
Other medical practitioners	Psychiatrist	3.510	0.063
Other medical practitioners	Psychiatrist and psychotherapist	3.000	0.146
Psychologist and psychotherapist	Psychiatrist	−6.924	**< .001**
Psychologist and psychotherapist	Psychiatrist and psychotherapist	−2.247	0.385
Psychiatrist	Psychiatrist and psychotherapist	0.800	0.942

As for the politeness-related features, contingency table analyses revealed no significant gender-based differences in the use of greetings (χ²(1, N = 100) = 0.0601, p = .806) and appellations (χ²(1, N = 100) = 2.58, p = .108). However, a statistically significant association was found for expressions of encouragement and/or best wishes (χ²(1, N = 100) =4.09, P = .043), suggesting that the difference in the frequency of use is related to gender, although the effect size was almost low (Cramer’s v = 0.202). Specifically, female practitioners were more likely to include wishes of good luck or encouragement in their responses (see Table 10).

**Table 10:** Gender-based differences in practitioners’ use of expressions of encouragement, best wishes, and availability declarations.

	Gender		
Practitioners’ expressions of encouragement and/or best wishes, declarations or availability	Female	Male	Tot.
Absent	23 (69.7%)	58 (86.6%)	81 (81%)
Present	10 **(30.3%)**	9 (13.4%)	19 (19%)
**Tot.**	33 (100%)	67 (100%)	100 (100%)

No statistically significant gender-based difference was found for pragmatic alignment (χ²(2, N = 148) = 4.50, p = .105), although female practitioners show a higher rate of alignment (65.3%, i.e. 32 aligned replies out of 44 total) compared to male practitioners (48.5%, i.e., 48 aligned replies out of 103 total).

No statistically significant association was also found between gender and empathic alignment (χ²(1, N = 100) = 0.248, p = .619). Nevertheless, women appeared to be more frequently empathically aligned (54.5%, i.e., 18 out of 33 replies) compared to men (49.3%, i.e., 33 out of 67 replies). Taking into account practitioners’ job specialisation, the contingency table analysis revealed no statistically significant differences in the use of greetings (χ²(3, N = 100) = 2.02, p = .568), appellations (χ²(3, N = 100) = 5.21, p = .157), and expressions of encouragement, best wishes, or availability (χ²(3, N = 100) = 5.38, p = .146). Similarly, no significant association was also found between practitioners’ job specialisation and pragmatic alignment (χ²(6, N = 148) = 5.95, p = .429). All professionals, regardless of their specialisation, tend to produce aligned responses more frequently than misaligned or partially aligned ones. Other medical practitioners displayed full alignment in all their responses (4 out of 4). Among psychiatrists, 55.6% of the replies were aligned (35 out of 63), while psychiatrists with psychotherapeutic training reached 80% alignment (4 out of 5). There was a more balanced distribution among psychologists and psychotherapists, but aligned responses still represented the largest proportion at 48.7% (37 out of 76).

No significant association was also found between practitioners’ job specialisation and empathic alignment (χ²(3, N = 100) = 0.470, p = .925).

#### Comparison.

The analysis carried out to identify gender-related differences in the two corpora revealed several similarities, but also some differences. In terms of politeness structures, no significant differences were found in IAC, while in PAC the only statistically significant difference concerns the greater use of expressions of encouragement and helpfulness by women. In terms of pragmatic alignment, although there are no statistically significant differences, women seem to be more aligned in both IAC and PAC. In PAC, this also applies to empathic alignment.

However, the analysis carried out to identify differences related to job specialisation shows more pronounced differences between the two corpora. While no statistically significant differences were found in PAC for either polite expressions or pragmatic and empathic alignment, several statistically significant differences were found in IAC for greetings and expressions of encouragement and helpfulness, which were used more frequently by psychologists and psychotherapists, and for appellatives, which were used more frequently by the ‘other doctors’ category. Although no statistically significant differences were found for pragmatic and empathic alignment, in IAC pragmatic alignment appears to be greater among psychiatrists and other doctors, while empathic alignment appears to be greater among psychologists and psychotherapists.

## 4. Some linguistic examples

Selected examples of Q&A interactions from IAC and PAC are discussed below.

*IAC*

***(1) IAC-12***

**Q:** *Salve, vorrei sapere perché in prossimità del tramonto quando arriva il buio, mi sale l’ansia senza un apparente motivo provocandomi difficoltà respiratoria e sensazione di pressione sul petto. Che ne pensate?*

Hello, I would like to know why, when it gets dark, I get anxious for no apparent reason, causing me difficulty breathing and a feeling of pressure on my chest. What do you think?

**R:** *Salve, mi spiace molto per la situazione che descrive poichè comprendo il disagio che può sperimentare e quanto sia impattante sulla sua vita quotidiana. Ritengo fondamentale che lei possa richiedere un consulto psicologico al fine di esplorare la situazione con ulteriori dettagli, elaborare pensieri e vissuti emotivi connessi e trovare strategie utili per fronteggiare i momenti particolarmente problematici onde evitare che la situazione possa irrigidirsi ulteriormente. Credo che un consulto con un terapeuta cognitivo comportamentale possa aiutarla ad identificare quei pensieri rigidi, disfunzionali e maladattivi che le impediscono il benessere desiderato mantenendo la sofferenza in atto e possa soprattutto aiutarla a parlare con se stesso/a utilizzando parole più costruttive. Credo che anche un approccio EMDR possa esserle utile al fine di rielaborare il materiale traumatico connesso ad eventi del passato che possono aver contribuito alla genesi della sofferenza attuale. Resto a disposizione, anche online. Cordialmente, dott XXX*

Hello, I am very sorry for the situation you describe as I understand the discomfort you may be experiencing and how it is impacting on your daily life. I think it is essential that you seek psychological counselling in order to explore the situation in more detail, to process related thoughts and emotional experiences, and to find useful strategies for coping with particularly problematic moments in order to prevent the situation from becoming even more rigid. I think that a consultation with a cognitive-behavioural therapist might help you to identify those rigid, dysfunctional and maladaptive thoughts that prevent you from achieving the desired wellbeing by keeping the suffering going and can above all help you to talk to yourself using more constructive words. I think that also an EMDR approach may also be helpful to you in order to reprocess traumatic material related to past events that may have contributed to the genesis of her current suffering. I remain at your disposal, also online. Cordially, Dr XXX. The interaction in extract (1) is a clear example of a pragmatic and empathic misalignment. In the opening post, after a polite greeting, the patient asks two questions: the first is indirect (I *would like to know why...*), aimed at understanding the causes of a set of symptoms he describes (At sunset he gets anxious, has difficulty breathing, and feels pressure in his chest); the second is direct (*What do you think?*), aimed at finding out the opinions of experts regarding the symptoms described. The patient does not show any orientation towards emotional support, he does not seek empathic understanding; he presents himself as an information-seeker, rather than an empathy-oriented seeker. Nonetheless, the psychotherapist’s response – which opens and closes with rather formulaic polite expressions and offers of availability – is pragmatically and empathically misaligned. Indeed, from a pragmatic perspective, the practitioner does not address the patient’s questions, but, on the contrary, offers a series of unsolicited advice (*I think it is essential that you seek psychological counselling*; *a consultation with a cognitive-behavioural psychotherapist might help you*; I *think that also an EMDR approach may also be helpful*), which can be problematic from a dialogic and relational perspective [35]. From an empathic point of view, the response is equally misaligned. In fact, the practitioner expresses both affective (‘I am very sorry’) and cognitive (‘I understand the discomfort’) empathy, even though the patient did not request or solicit any empathic response in his post. Therefore, the psychotherapist’s reply can be considered as an empathically misaligned unsolicited reply.

***(2) IAC-27***

**Q:** *Scusate volevo* sapere *se ci sono controindicazioni nel prendere eutimil e Xanax. Grazie*

Sorry I wanted to know if there are any contraindications to taking eutimil and Xanax. Thank you

**R:** *No, è* un’associazione *frequente.*

No, it is a frequent association.

The exchange in excerpt (2) is brief and content-focused. The patient’s post, which opens with *Scusate* (‘Sorry’) instead of a proper greeting, contains an indirect hypothetical question introduced by a formula typical of informal spoken Italian: the use of the imperfect tense of courtesy (*volevo sapere* [I wanted to know]) instead of the standard Italian *vorrei sapere* [I would like to know]. It is a request for information about the possible contraindications of taking two medications (an anxiolytic and an antidepressant) together. The post ends with a *thank you*. The response given by the practitioner – in this case, a psychiatrist – is very concise and lacks politeness markers. However, it is pragmatically aligned, as it addresses the patient’s request for information, and it is also empathically aligned, as it mirrors the emotionally neutral tone of the patient’s message with an equally neutral reply.

***(3) IAC-29***

**Q:** *Buonasera, sono un ragazzo di xx anni e da qualche mese soffro di depressione reattiva, sono sempre triste, a volte non ho voglia neanche di lavarmi e il mio psicoterapeuta mi ha prescritto un antidepressivo Sereupin, sono un po’ titubante nel prenderlo e volevo sapere se davvero è utile nella mia condizione e in quanto tempo fa effetto e se dovrò passare tanto tempo a fare uso di questo farmaco. Grazie mille in anticipo*

Good evening, I’m a xx year old guy and for the last few months I’ve been suffering from reactive depression, I’m always sad, sometimes I don’t even want to wash myself and my psychotherapist has prescribed me an antidepressant Sereupin, I’m a bit hesitant about taking it and I wanted to know if it really is useful in my condition and how long it takes to take effect and if I’ll have to spend a long time using this drug. Thank you very much in advance

**R:** *Salve, mi dispiace molto per come si sente, ma la paroxetina, principio attivo del sereupin, è un farmaco molto studiato e collaudato! Purtroppo non so consigliare senza prima conoscerci meglio se è un farmaco che può fare o meno al caso vostro, ma dalla sintomatologia descritta sembrerebbe indicato e potrebbe farla sentire meglio. In genere funziona bene dopo le prime tre settimane di trattamento, e l’assunzione deve essere prolungata fino alla risoluzione del quadro clinico e in alcuni casi anche oltre per garantire un mantenimento di un buon tono dell’umore (in media, nei casi classici di depressione, si può valutare la sospensione dopo 6 mesi liberi dai sintomi). Se accusa effetti collaterali o ha dei dubbi la invito comunque a parlarne con il vostro terapeuta, che saprà sicuramente consigliare per il meglio! Saluti*

Hello, I am very sorry for how you feel, but paroxetine, the active ingredient in sereupin, is a very well researched and tested drug! Unfortunately I can’t advise without first getting to know each other better whether or not it is a drug that may or may not be right for you, but from the symptoms described it would seem to be indicated and may make you feel better. It generally works well after the first three weeks of treatment, and the intake should be continued until the clinical picture resolves and in some cases even beyond that to ensure that a good mood is maintained (on average, in classic cases of depression, discontinuation can be considered after 6 months symptom-free). If you experience any side effects or have any doubts, however, I invite you to talk to your therapist, who will certainly be able to advise you for the best! Greetings

In excerpt (3), the user’s post opens with a greeting (*Good evening*), immediately followed by a series of details about his gender, age, diagnosis, emotional state, and the treatment (specifically, an antidepressant) prescribed by their ‘psychotherapist’. However, the user expresses hesitation about this therapy and asks a series of indirect questions, again introduced by the informal phrase ‘*volevo sapere*’ (I wanted to know), designed to elicit both information and confirmation. The first is a hypothetical question through which the user seeks confirmation of the actual usefulness of the drug in his condition; the second concerns the time required for the medication to take effect; the third – another hypothetical question – concerns the expected duration of the treatment. The post ends with the polite closing formula ‘*Thank you very much in advance*’. Although the main aim of the message is to gather information/confirmation, it is also emotionally charged (e.g., ‘*I am always sad, sometimes I don’t even feel like washing myself*’; ‘*I am hesitant*’).

The response from the practitioner, a psychiatrist, includes greetings both at the beginning (*Salve* [Hello]) and at the end (*Saluti* [Greetings]), and can be described as both pragmatically aligned (as it provides detailed answers to all of the patient’s questions) and empathically aligned (as it conveys both regret and understanding for the interlocutor’s emotional state, and attempts to offer reassurance – as far as possible given the lack of direct clinical knowledge – about the potential effectiveness of the treatment). In particular, the presence of exclamation marks, which are somewhat unusual in written medical consultations, appears to be intended to express reassurance and encouragement, including in relation to the patient’s trust in their therapist.

***(4) IAC-13***

**Q:** *Ho l’ansia super fortissima cosa posso fare? grazie rispondetemi*

I have super strong anxiety what can I do? thank you answer me

**R:** *Salve, questi superlativi rendono proprio l’idea di quanto sia forte la sua ansia. Ha fatto bene a scrivere. Ci tengo a rassicurarla dicendole che essendo l’ansia un’emozione, non può durare per sempre, ma è solo transitoria. Se quest’ansia tende a tornare, l’ideale sarebbe effettuare una terapia psicologica. L’approccio più efficace per l’ansia è quello cognitivo-comportamentale, che io utilizzo. Se vuole posso riceverla in un incontro per valutare la sua problematica più nel dettaglio e poi sceglierà se proseguire. A disposizione, Dott.ssa XXXXX*

Hi, these superlatives really convey how strong your anxiety is. You did well to write. I want to reassure you that since anxiety is an emotion, it cannot last forever, but is only temporary. If this anxiety tends to return, the ideal would be to undergo psychological therapy. The most effective approach for anxiety is the cognitive-behavioural one, which I use. If you like, I can meet with you to assess your problem in more detail and then you can decide whether to proceed. Yours sincerely, Dr XXXXX.

Excerpt 4 is an example of both pragmatic and empathic alignment. The patient’s distress is clearly evident in the use of the opening statement ‘*I have very strong anxiety’.* Equally clear seems to be her urgency to receive an answer to her direct request (*what can I do?*), an urgency that is evident both in the use of the imperative ending (*answer me*) – although anticipated by thanks (*thank you*) – and in the intrinsic brevity of the post and an incorrect use of punctuation. Although there is no explicit request for emotional support, it seems to be implied by the content and tone of the post which, at the same time, is pragmatically configured as a request for advice (*what can I do?*) and emotionally as a request for support. The psychotherapist’s response begins with a formal greeting and a clear expression of empathic understanding (*these superlatives really convey the extent of your anxiety*), followed by a supportive acknowledgment that reaching out was the right thing to do, and a reassuring message (*I want to reassure you*), emphasising the transient nature of anxiety. In response to the patient’s explicit request (*what can I do?*), the psychotherapist provides advice in a mitigated form, beginning with a hypothetical sentence: *if this anxiety tends to return*, *the ideal would be to undergo psychological therapy*. She then suggests the cognitive-behavioural approach – presented as the most effective and which she personally uses – and offers her availability for an initial evaluation. The patient, who is looking for both advice on what to do and for empathetic reassurance, finds in the psychotherapist a complementary figure who not only shows her empathetic understanding and gives her the advice she needs, but is also willing to help her concretely.

*PAC*

***(1) PAC-77***

**Q:** *Czy zbyt mała dawka velafaksyny może być nie skuteczna w leczeniu depresji? I czy po zwiększeniu dawki do 150 mg muszę czekać 2 tygodnie aż zacznie działać?*

Can too low a dose of velafaxine not be effective in treating depression? And do I have to wait 2 weeks for it to take effect after increasing the dose to 150 mg?

**R:** **Witam, przy dawce <150mg może nie ujawniać się pełny efekt terapeutyczny wenlafaksyny. Po zwiększeniu dawki ww. leku do 150 mg przez lekarza psychiatrę należy poczekać min. 2tyg, zanim nastąpi poprawa stanu psychicznego.**

Hello, at a dose of <150mg the full therapeutic effect of venlafaxine may not be visible. After increasing the dose of the above-mentioned drug to 150 mg by a psychiatrist, you should wait min. 2 weeks before the mental state improves. Regards

Excerpt 1 is an example of pragmatic and non-empathic alignment. Both the question and the reply are concise. The patient is clearly information seeking, asking two direct questions about a specific medication used to treat depression. The direct nature of the inquiry is also evident in the absence of any politeness acts, such as a greeting or an acknowledgement. In his brief response, which begins and ends with conventional greetings, the psychiatrist answers both questions and provides information about the therapeutic dose of the medication and the time needed to observe its effects. Although the use of mitigation can be found in the response (*may not be visible*), with an additional implicit suggestion that the dose needs to be increased by a psychiatrist, the reply addresses the patient’s request and provides the information sought. The question and the response can also be considered as non-empathic but aligned, as both messages are emotionally neutral and neither the patient nor the doctor appears to be seeking and/or offering empathy or emotional support.

***(2) PAC-75***

**Q:** **Mam zaburzenia lękowe Boję się chodzić po chodniku i po jezdni Moje nogi stają* *się sztywne Poruszam się podpierając się kijkami Jedynie na trawie mogę chodzić w miarę swobodnie Biorę leki przeciwdepresyjne i przeciwlękowe ale niewiele pomagają* *Co mam robić? Czy jest nadzieja na wyzdrowienie?**

I have anxiety disorders I am afraid to walk on the pavement and on the road My legs become stiff I move with sticks Only on grass can I walk fairly freely I take antidepressants and anti-anxiety medication but they don’t help much What should I do? Is there hope for recovery?

**R:** *Oczywiście, że jest nadzieja. Rozumiem, że zaburzenia lękowe stwierdził psychiatra, który przepisał leki – pytanie od jak dawna są* *przyjmowane. Być może nie rozwinęły jeszcze w pełni swojego działania (np. leki przeciwdepresyjne potrzebują* *na to zwykle kilku tygodni). Poza tym gorąco zachęcam do podjęcia psychoterapii. Pomoże ona poradzić sobie z napięciem i przy wsparciu terapeuty stopniowo przezwyciężyć lęk, a także zrozumieć gdzie leży jego przyczyna. Życzę powodzenia!*

Of course there is hope. I understand that the anxiety disorder was diagnosed by the psychiatrist who prescribed the medication – the question is how long they have been taken. Perhaps they have not yet fully developed their effects (antidepressants, for example, usually take a few weeks to do so). Other than that, I would strongly encourage you to undertake psychotherapy. It will help you to cope with the tension and, with the support of a therapist, gradually overcome the anxiety and understand where the cause lies. I wish you the best of luck!

Excerpt 2 offers another example of pragmatic and empathic alignment. In their message, the patient describes the disorder, including the emotional and physical symptoms which they suffer from, adding that they haven’t found much relief from medication. The statement is followed by two questions, in the first of which the patient asks for advice on the actions that they can take (*what should I do?*), while in the second they ask whether there is hope of recovery, which can be interpreted as an implicit request for reassurance or emotional support. The psychologist and psychotherapist’s response begins with an emphatic expression of support reassuring the patient that recovery is possible (*of course there is hope*). She then offers mitigated assessment of the patient’s problems (*I understand*; *perhaps*; *usually*), and proceeds to suggest psychotherapeutic treatment, explaining how it might help the user with their disorder. The reply concludes by wishing the patient well. Since the doctor provides both advice and emotional support which the user is seeking, the question and answer can be considered as pragmatically and empathically aligned.

***(3) PAC-94***

**Q:** *Mam xx lat. Od kilku lat zmagam się z problemem nadmiernej potliwości głównie na twarzy. Zauważyłem, że* **Q:** *występuje to tylko w sytuacjach stresujących. Podczas pobytu w kościele, na zakupach, na spotkaniu ze znajomymi.. nie potrafię przez to normalnie funkcjonować. Utożsamiam to z problemem który opisują* *w internecie jako “agorafobia”. Stresujac się poci mi się twarz, przez co stresuje się jeszcze bardziej itd itd. błędne koło. Kiedy się nie stresuje nicz tych objawów nie wystepuje. Co gorsza we wrześniu planujemy z narzeczoną* *ślub.. jestem tym faktem przerażony, a w zasadzie tym jak zareaguje. Co mogę z tym zrobić? Dodam, że próbowałem kuracji botoksem i przyniosło to raczej marny efekt.*

I am xx years old. I have been struggling with the problem of excessive sweating mainly on my face for several years. I have noticed that this only occurs in stressful situations. While in church, shopping, meeting friends.... I can’t function normally because of it. I identify it with a problem they describe on the internet as ‘agoraphobia’. When I get stressed my face sweats, which makes me even more stressed, etc. etc. A vicious circle. When I am not stressed none of these symptoms occur. To make matters worse, my fiancée and I are planning our wedding in September and I am terrified of how I will react. What can I do about it? I have tried botox treatment and it had a rather poor effect.

**R:** *Zaleciłabym przede wszystkim terapię w nurcie behawioralno-poznawczym.Do rozważenia objawowo farmakoterapia.*

I would recommend first and foremost behavioural-cognitive therapy. For consideration pharmacotherapy as a supportive symptom.

Extract 3 is an example of a pragmatically aligned but empathically misaligned exchange. The patient’s message begins by stating his age, followed by a description of the problem he has been suffering from, with a detailed account of the symptoms and the circumstances in which they occur, the patient’s self-diagnosis, and the forms of treatment he has implemented. The description is followed by an explicit request for advice (*What can I do about it?*), in which the patient asks about possible measures that can be taken to solve the problem. The message does not contain an explicit request for emotional support. However, its emotional tone can be inferred from the description of the patient’s suffering, his feelings and experiences (*I’ve been struggling with*; *I can’t function*; *I’m terrified*; *a vicious circle*; *to make matters worse...*), as well as from the somewhat chaotic form of the description, with numerous spelling mistakes and the use of ellipsis. In her rather brief response, the psychiatrist offers mitigated advice (*I would recommend*; *for consideration*), suggesting that the patient undergo behavioural-cognitive therapy, accompanied by pharmacotherapy. What is also noticeable is the absence of any acts of conventional politeness, such as, for instance, greetings. Since the doctor gives the patient the advice he is seeking, the response can be considered as pragmatically aligned. However, the reply is an examples empathic misalignment because of its neutral tone and the lack of any form of empathetic expression offered in response to the patient’s emotionally charged message.

***(4) PAC-41***

**Q:** *Osoba, z którą* *jestem cierpi na fobię społeczną, depresje i ataki silnej paniki. Jak pomóc tej osobie gdzie często apatia spowodowana silnymi lekami uniemożliwia jej czynności, stres i rozchwianie emocjonalne jest wysokie a napady lękowe są* *silne i często podręcznikowe działania nie pomagają? Co robić w momentach krytycznych, jakie zadawać pytania aby czuła się bezpiecznie? Jak przy tym dbać o siebie nawzajem aby relacja przetrwała?*

The person I am with suffers from social phobia, depression and severe panic attacks. How do I help this person where often apathy due to strong medication prevents her from doing her activities, stress and emotional upset are high and the anxiety attacks are strong and often textbook measures do not help? What to do in critical moments, what questions to ask to make her feel safe? At the same time, how do we take care of each other so that the relationship survives?

**R:** *Na wszystkie postawione wyżej pytania, czeka odpowiedź* *w gabinecie psychoterapeutycznym.*

All the questions raised above are waiting to be answered in the psychotherapeutic office.

The user’s message begins with a statement of their problem (*The person I am with suffers from...*), followed by a series of direct questions, in which the person seeks advice and information about actions that can be taken to alleviate problems and to help their partner to feel safe and to save their relationship (*How do I help...*; *What to do...*; *what questions to ask...*; *how do we take care...*). The user lists different disorders the person suffers from (*social phobia, depression and severe panic attacks*) and describes problems with treatment (*strong medication prevents her from doing her activities*; *textbook measures do not help*). The message is emotionally charged, although there is no explicit request for empathy. The emotional tone is reflected in the description of the severity of the symptoms and problems (*severe panic attacks*; *stress and emotional upset are high*; *anxiety attacks are strong*). In his reply, the psychologist and psychotherapist gives a concise one-sentence answer stating that *all the questions are waiting to be answered in a psychotherapeutic office*. The practitioner does not address any of the user’s questions, does not suggest any solutions, measures or forms of treatment aiming to alleviate the problem. Nor does he offer any expression of empathy, emotional support or understanding, his message being rather impersonal and dismissive in tone. The impersonal nature of the reply is reinforced by the absence of any politeness markers. The user’s question and the doctor’s reply can thus be interpreted as both pragmatically and empathically misaligned.

## 5. Discussion

In this study, we conducted a cross-cultural (Italian – Polish) analysis of online health communities involving patients and healthcare professionals on the international multi-service platform *MioDottore* (Italy) *ZnanyLekarz* (Poland). The primary objective was to explore similarities and differences in the communication patterns of users experiencing anxiety and the healthcare professionals responding to their queries. Two comparable corpora were selected: the Italian Anxiety Corpus (IAC) and the Polish Anxiety Corpus (PAC). These datasets were similar in terms of key characteristics, including the number of question-answer pairs (100), the average length of the posts (with healthcare professionals generally providing shorter answers compared to patients’ questions), and the gender distribution of healthcare professionals (a higher proportion of male professionals in both corpora). However, there was a difference between the datasets when it comes to professional specialisation of the experts most frequently offering their replies: in the IAC, responses were more often provided by psychiatrists (including those with psychotherapeutic training), whereas in the PAC, psychologists and psychotherapists were the main respondents.

The quantitative analysis, as well as the analysis of some excerpts from the two corpora revealed several similarities and differences.

**RQ1 (Communication Patterns of Patients)** With respect to the first research question, we observed some differences. In PAC, patients predominantly used direct questions (83.1% of the total number of questions), while in IAC, in addition to direct questions (66.7% of the total number of questions), they also resort to indirect questions (25.2% of the total number of questions). The results confirm a preference for directness in requests among Polish speakers in contrast to the speakers of other languages, as shown in other studies [36–37]. As far as pragmatic functions are concerned, in both cases users ask questions aimed at obtaining information, opinions and advice. This suggests that, even when dealing with psychological issues, such as anxiety, people mainly use online health communication to obtain practical information rather than as a space for venting or self-disclosure. This does not imply – as discussed above – that users’ posts are lacking in emotional components or in explicit or implicit appeals for emotional support. Rather, such elements are frequently combined with requests for practical information and only rarely occur in posts devoted exclusively to venting or self-disclosure. However, a significant difference was observed in the use of greetings, forms of address (appellations), and acknowledgments: while Italian users resort to them more often, Polish users make considerably less use of them, showing a preference for more direct and straightforward posts. Regarding such politeness formulae, while their use is typical of formal face-to-face doctor-patient interaction in both Italian and Polish, the transfer of these conventions to the online context seems to be more common among Italian users. For Polish speakers, the mediated and anonymous form of the interaction seems to encourage greater directness and less attention to conventionalized politeness. It may also be the case that among Polish users, the urgency of receiving a response – probably linked to the nature of the condition for which they are seeking help (i.e., anxiety) – appears to take precedence over the use of polite expressions, typically expected in a formal, even if online, setting [38–39]. On the contrary, emotional components appeared with similar frequency in both corpora.

**RQ2 (Communication Patterns of Healthcare Professionals)** Regarding the second research question, we found more similarities than differences between the corpora. Firstly, in both datasets, psychologists and psychotherapists were found to produce longer written responses compared to other healthcare professionals, likely reflecting their training, which places a strong emphasis on verbal expression and therapeutic dialogue. Furthermore, greetings were used by 70% of Italian healthcare professionals and 65% of Polish ones, while expressions of encouragement appeared in approximately 20% of posts in both corpora. The use of emotionally charged language was also comparable (27% in IAC and 20% in PAC). Pragmatic alignment with user requests was slightly higher in IAC (89%) than in PAC (80%), but empathic alignment was identical (51% in both corpora). Nonetheless, in both datasets, empathic misalignment occurred in nearly half of the interactions. The relatively high degree of pragmatic alignment and the rather low occurrence of emotional components in the replies indicate a more transactional orientation of the messages, and a greater focus on providing the requested information and advice rather than on establishing rapport. In other words, our data suggest that many practitioners maintain a professional distance, rather than respond to users’ emotional cues or express empathy explicitly. The overall interactive style appears to prioritise problem-solving over establishing relational connections, even in contexts related to mental health disorders such as anxiety, where a different approach might be expected. The mismatch between users’ potential need for emotional validation and practitioners’ focus on the problem may be a broader characteristic of digital health communication, where efficient transmission of information often seems to take precedence over attention to the emotional component. These results seem to be consistent with those of other studies [e.g., 40] conducted in other contexts, according to which, while digital technologies can improve the efficiency of care, they may also undermine the relational depth and emotional presence that are central to caring roles. The weak attention to the emotional components can be due to several reasons, including the nature of the interactions (which are remote, text-based, occurring between people who do not know each other, and taking place within a specific context, such as that of multi-service healthcare platforms), which can make both the recognition of emotional support requests and the demonstration of such support (i.e., the empathic behaviour) challenging, the fact that focusing on practical issues is less demanding for healthcare professionals (both psychologically and in terms of time) than also addressing emotional components, and insufficient training on these aspects.

As for politeness strategies, the relatively frequent use of these formulae suggests that most experts tend to transfer the conventions typical of formal face-to-face interactions in Italian and Polish.

**RQ3 (Impact of Gender and Professional Specialisation)**. With respect to the third research question, no gender-based differences were observed in the use of politeness features among IAC professionals. However, in PAC, female practitioners more frequently used encouraging, supportive, and well-wishing expressions. While pragmatic and empathic alignment showed no statistically significant gender-based differences, there was a slight tendency for female professionals to demonstrate a higher degree of pragmatic alignment in both corpora, and a higher degree of empathic alignment, particularly in PAC. These results seem to suggest that Polish female experts show a greater orientation towards supportiveness, cooperation, rapport and emotional expressivity, i.e., features associated with women’s conversational style in previous studies [41–42]. The findings confirm previous research contrasting gender-specific language behaviour of Polish men and women, which found a greater tendency towards affective expression among women [43]. The data for IAC, on the other hand, are consistent with previous research conducted on Italian medical students, which suggests that empathy – particularly in its cognitive dimension, involving the ability to put oneself in another’s shoes – may not be influenced by gender [44]. However, they contradict other studies indicating that empathy levels are higher among female medical students [45].

In contrast to what was observed for gender, significant differences were found in IAC as far as professional specialisation is concerned. Psychologists and psychotherapists were more likely to use greetings, encouragement, and supportive expressions, while appellations were used exclusively by non-psychiatrist doctors. Regarding pragmatic alignment, in IAC, psychiatrists’ (including those with psychotherapeutic training) and non-psychiatrist doctors’ answers demonstrated greater relevance to users’ requests (for information and advice). While empathic alignment did not differ significantly by specialisation, there was a clear trend in IAC showing that psychologists and psychotherapists were generally more empathic than psychiatrists and other doctors, probably due to their training formation.

While minor, non-significant differences in attention to emotional aspects were observed in both corpora – particularly among certain professional groups and among women – our results, consistent with other research, highlight the need of further training and education [46] to equip healthcare professionals in general, and those operating remotely in particular, with empathy skills and to foster the development of what some researchers refer to as “virtual compassion competencies” [47].

Although this study has brought to light a range of interactional phenomena that have not previously been explored (at least not from a contrastive perspective), the main limitation lies in the relatively small size of the two corpora, which prevents broad generalisations. Future research should aim to expand the datasets to allow for more robust quantitative analyses and more reliable findings.

Given the differences that emerged regarding both the gender and professional specialisation of practitioners, future studies could take two complementary directions: a deeper investigation of comparable interactions in offline, face-to-face medical settings, and an examination of whether similar patterns are replicated also in healthcare interactions involving AI-based chatbots. Exploring these latter dynamics could offer valuable insights into how digital health communication is evolving and what implications this has for the design of more effective, empathetic, and culturally sensitive AI systems. This will be the focus of our next phase of research.

### Declaration of generative AI and AI-assisted technologies in the writing process

During the preparation of this work the authors used Deepl and ChatGPT 4.0 to check grammar, spelling and generally to improve the English language. After using this tool/service, the authors reviewed and edited the content as needed and take full responsibility for the content of the publication.

## Supporting information

S1 Table Tagging scheme for Italian and Polish online anxiety consultations. (DOCX)
