# Supplementary material for: Communicative patterns in online health communities: A comparative study of Italian and Polish
Source: PLoS One. 2025 Sep 23;20(9):e0333011. doi: 10.1371/journal.pone.0333011 (PMC12456825; doi:10.1371/journal.pone.0333011)
Supplement: S1 Table — (DOCX) [file pone.0333011.s001.docx]

**Table 1: Tagging scheme for Italian and Polish online anxiety consultations**

|  | ***Examples for IAC*** | ***Examples for PAC*** |
| --- | --- | --- |
| **USERS** |  |  |
| ***Types of questions*** |  |  |
| **direct question/request**  (i.e., questions marked by a question mark or that, even in the absence of question marks, have the structure of direct questions) | *Può l'assunzione puntuale provocare dipendenza?* [Can regular intake cause addiction?] | *Czy mogę brać pramolan?* [Can I take pramolan?] |
| **indirect question/request**  (i.e., questions that do not require a question mark and are usually introduced by ‘verba dicendi’ (e.g., / I was wondering whether …) or by knowledge verbs (e.g., I wanted to know if…). Indirect questions often use the conditional or past indicative to convey polite requests. | *volevo chiedere se continuare dalla psicologa o no* [I wanted to ask whether to continue with the psychologist or not] | *Chciałabym się dowiedzieć czy te leki nie wchodzą w interakcję* [I would like to know if these medicines do not interact] |
| **mixed form** (i.e., those forms generally introduced by an expression of not knowing followed by a question or by a request) | *Non so cosa devo prendere. In casa ho Xanax. Va bene questo medicinale?* [I don't know what I should take. I have Xanax at home. Is this medicine OK?] | *Jednak nie wiem czego to może być przyczyną. Gdzie szukać pomocy?* [however I don’t know what this could be the cause of. Where to seek help?] |
| **implicit question/request** (i.e., instances where no question marks are used, but patients express a lack of knowledge or certainty; statements that are interpreted as questions or requests by doctors, who typically respond by addressing the epistemic gap) | *Non so se c'entri il cambio farmaco o se è solo una coincidenza* [I don't know if it has something to do with the change in medication or if it's just a coincidence] | *Nie wiem czy wzrost hormonów nie zagłuszył tabletek* [I don’t know if the rise in hormones hasn’t drowned out the pills] |
| **no_question** (i.e., posts that do not contain explicit questions or requests, but are nonetheless interpreted as such by doctors, who respond) | *Salve mi hanno trovato il cuore ingrossato e pompa poco sangue rischio la vita ,poi faccio una cura di atenol e diuretici grazie* [Hi, they found an enlarged heart and poor blood flow in my heart, it's life threatening, so I'm taking atenolol and diuretics. Thank you] | *Witam ,lekarz psychiatra przepisał mi mozarin na nerwicę ,źle się po nim czuje ,mam zawroty głowy i boli mnie głowa ,rano nie mogę nic jeść.Czuje się jeszcze gorzej niż przed podaniem leku.* [Hello, my psychiatrist has prescribed mozarin for my neurosis, I feel bad after it, I feel dizzy and have headaches, in the morning I can't eat anything, I feel even worse than before the drug.] |
|  |  |  |
| ***Questions pragmatic functions*** |  |  |
| requests for information/opinion/confirmation | *se continuo a prenderlo potrei avere dei problemi ?* [If I continue to take it, could I have problems?] | *Czy te leki można łączyć?* [Can these medicines be combined?] |
| requests for help/advice/reassurance | *Cosa mi consigliate?* [What do you recommend?] | *Proszę o pomoc* [Please help me] |
| venting/disclosure | *Soffro di ansia da circa xx anni curata con antidepressivi e ansiolitici...da circa x anni prendo paroextina...e al bisogno alprazolam .... ultimamente ho dovuto aumentare l'alprazolam.... perché paura di tutto e penso continuamente a questo* [I suffer from anxiety for about xx years treated with antidepressants and anxiolytics ... for about x years I take paroxetine ... and if necessary alprazolam .... lately I had to increase the alprazolam .... because I am afraid of everything and I constantly think about this] | *Witam ' aktualnie mam xx lat i zmagam się z pewnym problemem , a dokładniej chodzi o to że nie potrafię opuścić domu rodzinnego ponieważ odczuwam lęk przed zmianą otoczenia i utratą kontaktu z rodzicami . Chciala bym normalnie funkcjonować np wyjechać z chłopakiem zagranicę jednak kilka dni przed planowana data wyjazdu wpadam w panikę płacze , nie mogę jesc , mam problemy żołądkowe. Staram się nad tym panować jednak jest to silniejsze odemnie*  [Hello ‘ I am currently xx years old and I am struggling with a problem, namely that I am unable to leave my family home because I feel anxious about changing surroundings and losing contact with my parents. I would like to function normally, for example I would like to go abroad with my boyfriend, but a few days before I am supposed to leave I panic, cry, I can't eat and I have stomach problems. I try to control it, but it is stronger than me] |
| ***Greetings/Salutations*** (generally placed at the opening/closure of the post) | *Buongiorno* [Good morning] | *Witam* [Hello] |
| ***Appellations/Titles*** (i.e., the use of a title or form of address that is directed to the recipient of a message, often to signal recognition, respect, or to explicitly identify the addressee. | *Buongiorno dottori* [Good morning doctors] | ABSENT |
| ***Acknowledgements*** (i.e., forms of thanks, generally placed at the end of the post) | *Grazie in anticipo* [Thank you in advance] | *Dziękuję za odpowiedź* [Thank you for your answer] |
| **Emotional components** (i.e., terms expressing emotional states, often interpreted as requests for empathy) | *Salve (…), da x anno soffro d'ansia... da settembre va meglio ma mi è rimasta l'agitazione nel senso che ho sempre paura di avere un attacco […].* [Hi (...), **I've been suffering** from anxiety for a year… now things have got better since September, but I'm still restless in the sense that **I'm always afraid** of having an attack (...)]. | *Mam silne stany lękowe z atakami paniki... Ciągły strach o siebie, dziecko.. Zobaczę mały wyprysk na skórze i już przychodź strach* [I have severe anxiety with panic attacks.... **Constant fear** for myself, the baby.... I will see a small blemish on my skin and the **fear comes]** |
|  |  |  |
| **HEALTHCARE PRACTITIONERS** |  |  |
| **Greetings/Salutations** | *Salve* [Hi] | *Dzień dobry* [Good morning] |
| **Appellations/Titles** | *Salve Elisa* [Hi Elisa] | *Szanowny Panie* [Dear Sir] |
| **Acknowledgements** | ABSENT | ABSENT |
| **Expressions of encouragement and/or best wishes, declarations of availability** (i.e, polite forms of pre-closure) | *In bocca al lupo* [Good luck] | *Życzę powodzenia!* [Good luck!] |
| **Emotional components** (i.e. use of emotionally charged terms that act as an empathetic response) | *Salve Elisa, mi spiace molto per la situazione ed il disagio espresso e comprendo quanto possa essere difficile per lei convivere con questa situazione riportata.* [Hi Elisa, **I'm very sorry** for the situation and the discomfort you've expressed **and I understand how difficult it must be for you** to live with this reported situation.] | *To bardzo trudna sytuacja. Bardzo mi przykro, że to się Pani przydarzyło.* [This is a very difficult situation. **I am very sorry** this has happened to you.] |

The names of users and healthcare practitioners, as well as some temporal references, have been changed to fictitious ones or delated and replaced with xxx.
